# Salvage treatment strategies for refractory sudden sensorineural hearing loss—a comprehensive review and meta-analysis with practical recommendations

**DOI:** 10.3389/fneur.2025.1627892

**Published:** 2025-07-15

**Authors:** Piero Giuseppe Meliante, Luigi D’Avino, Giuseppe Barba, Edoardo Covelli, Carla Petrella, Christian Barbato, Antonio Minni

**Affiliations:** ^1^Department of Neuroscience, Mental Health and Sensory Organs (N.E.S.M.O.S.), Faculty of Medicine and Psychology, “Sapienza” University, Rome, Italy; ^2^Division of Otorhinolaryngology, Head and Neck Surgery, “V. Monaldi” Hospital, A.O.R.N. dei Colli, Naples, Italy; ^3^Department of Sense Organs (DOS), Institute of Biochemistry and Cell Biology (IBBC), National Research Council (CNR), Sapienza University of Rome, Rome, Italy; ^4^Department of Sense Organs (DOS), Sapienza University of Rome, Rome, Italy; ^5^Division of Otolaryngology-Head and Neck Surgery, Ospedale San Camillo de Lellis, ASL Rieti-Sapienza University, Rieti, Italy

**Keywords:** hearing loss, sudden, glucocorticoids/therapeutic use, intratympanic injection, hyperbaric oxygenation (HBO), treatment outcome

## Abstract

**Background:**

Sudden sensorineural hearing loss (SSNHL) affects up to 27 per 100,000 people annually, with more than half not regaining full hearing after following standard therapy. Identifying effective salvage treatments for refractory cases is critical to improve outcomes and reduce long-term auditory disability. This systematic review aims to assess the effectiveness of current salvage treatments for SSNHL unresponsive to first-line systemic corticosteroid therapy, and to develop an evidence-based treatment algorithm.

**Methods:**

A comprehensive search of PubMed, Embase, and Cochrane Library was performed for English language articles published between January 2010 and April 2025. Studies eligible for inclusion were clinical trials and large case series that evaluated salvage interventions in patients with SSNHL who were unresponsive to systemic therapy. Risk of bias was assessed using the risk of bias in non-randomized studies of interventions (ROBINS-I) tool.

**Results:**

A total of 41 articles met the inclusion criteria. Intratympanic steroids (ITS), including methylprednisolone and dexamethasone, showed consistent effectiveness, with methylprednisolone achieving better results (*p* < 0.05). Hyperbaric oxygen therapy (HY) was also effective, particularly at low frequencies. Combined ITS and HY yielded the best results in word recognition and pure-tone average (PTA) improvement, although they were not always statistically better than monotherapy. Early initiation of ITS was associated with improved outcomes, and ITS proved especially effective for high-frequency SSNHL and tinnitus (*p* = 0.002). Non-invasive therapies, such as constraint-induced sound therapy (CIST), have been promising in improving outcomes by decreasing maladaptive cortical reorganization. Additional emerging treatments [e.g., insulin-like growth factor 1 (IGF-1), urokinase, and surgical interventions] show potential but need further validation.

**Conclusion:**

ITS and HY, especially when combined, are the most effective salvage therapies for refractory SSNHL. Methylprednisolone may offer better outcomes than dexamethasone, and early intervention continues to be a crucial prognostic factor. CIST showed promising potential in reducing cortical maladaptation to sound deprivation.

**Systematic review registration:**

https://www.crd.york.ac.uk/PROSPERO/view/CRD42025645069, Identifier, CRD42025645069.

## 1 Introduction

Sudden sensorineural hearing loss (SSNHL) is marked by a rapid loss of hearing, often requiring urgent or emergency medical evaluation. It is clinically defined as a hearing loss of ≥30 dB across at least three or more frequencies occurring within a 72-h period (1). The annual incidence is 27 per 100,000 individuals in the United States. However, this data may be underestimated, as patients with mild symptoms or those who experience spontaneous recovery often do not seek medical attention (1, 2). Approximately 90% of cases are idiopathic; proposed etiologies are vascular compromise, viral infection, or autoimmune mechanisms (3). SSNHL can significantly impact a patient’s quality of life, and up to 50% of individuals may have a poor or no response to standard therapy (4–6).

Since the American Academy of Otorhinolaryngology-Head and Neck Surgery published its guidelines for SSNHL in 2019, several new studies have emerged, and various treatments have been explored for refractory SSNHL. These include standard intratympanic corticosteroids (ITS), hyperbaric oxygen therapy (HT), novel pharmacologic agents [e.g., insulin-like growth factor 1 (IGF-1), diuretics, urokinase, and migraine medications], surgical approaches, and targeted drug delivery systems (1, 7–11). Despite this growing interest, the optimal management strategy for patients unresponsive to first-line therapy remains unclear. Available evidence from comparative studies is sometimes heterogeneous (1, 12).

This systematic review aims to provide a comprehensive evaluation of treatments for SSHNL that are unresponsive to first-line steroid therapy, with the goal of clarifying the current therapeutic landscape and providing clinical decision-making support for this condition.

## 2 Materials and methods

This systematic review was conducted in accordance with the PRISMA guidelines and the Cochrane Handbook for Systematic Reviews of Interventions. The protocol was prospectively registered in the PROSPERO database under registration number CRD42025645069.

### 2.1 Search strategy

A systematic literature research was conducted in three databases: PubMed, Embase, and the Cochrane Library. The search strategy was summarized in Supplementary Table 1. Research query for articles regarding the treatment of adult (≥18 years old) patients affected by SSNHL with poor- or no-response to first-line therapy was used. We only considered English language articles published between January 2010 and April 2025.

### 2.2 Selection criteria

After removing duplicates, titles and abstracts were screened for relevance, followed by full-text review to determine eligibility. Studies were included if they evaluated therapeutic interventions for SSNHL that were refractory to first-line systemic corticosteroid therapy in adult populations. We included randomized controlled trials (RCTs), non-randomized clinical trials, and extensive case series, which we defined as those including a minimum of 20 patients treated with the intervention of interest. Case reports, systematic reviews, meta-analyses, animal studies, and studies focusing on pediatric populations were excluded. In cases where mixed etiologies of hearing loss were reported, studies were included only if data specific to idiopathic SSNHL were separately presented or could be extracted. This approach was taken to maintain consistency and improve the reproducibility of findings.

### 2.3 Risk of bias evaluation

**Figure 1:**
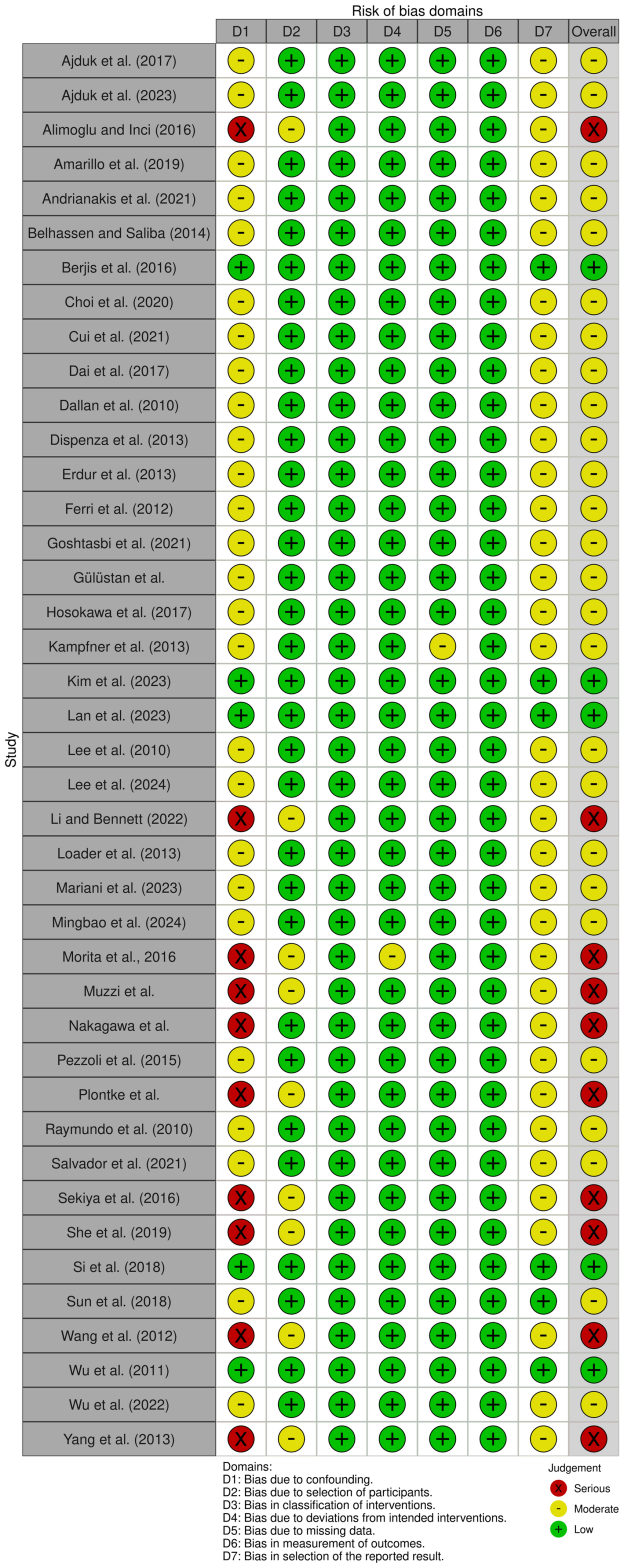
Risk of bias.

Eligible studies were assessed for risk of bias using the risk of bias in non-randomized studies of interventions (ROBINS-I) tool.^1^ Risk of bias outcomes were summarized in Supplementary Table 2 and were visually described using the ROBVIS tool (13) in Figure 1.

### 2.4 Data extraction

Reviewers collectively discussed the study outcomes after conducting independent analyses, and findings were shared. Data extracted from the included studies were synthesized narratively, and the risk of bias assessments were discussed in detail to critically evaluate the strength of evidence.

## 3 Results

**Figure 2:**
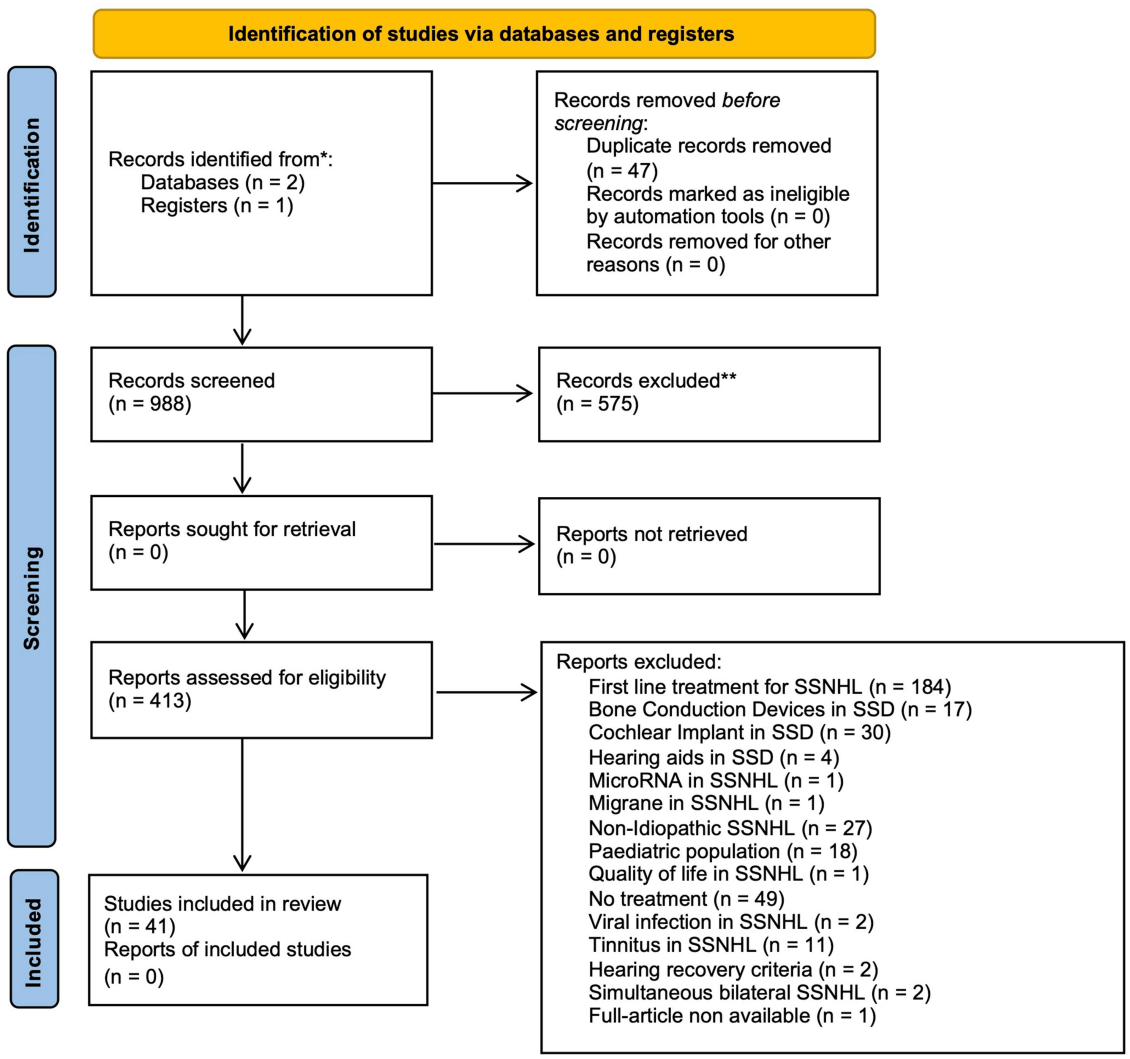
PRISMA flow diagram.

After article extraction and duplicate removal, 988 articles were identified. Article outcomes from each database were summarized in Supplementary Table 1. An initial screening of titles and abstracts led to the exclusion of 575 papers. Of the remaining 413 articles, 41 met the criteria of eligibility and were selected for inclusion in this systematic review. Figure 2 presents the PRISMA flow diagram, illustrating the database search process, screening steps, and reasons for exclusion of reports assessed for eligibility.

To provide a more precise and more comprehensive overview of the outcomes, we summarized the data from each study in the following sections, organized by intervention type.

### 3.1 Intratympanic corticosteroids

#### 3.1.1 Methylprednisolone

Amarillo et al. (14) conducted a retrospective study on 109 patients showing incomplete recovery after oral steroid therapy for SSNHL. The population was divided into two groups: 76 patients for the experimental group and 33 controls who refused to receive Intratympanic steroids (ITS). After 6 months, the treatment group demonstrated a mean pure-tone average (PTA) improvement of 10.84 dB compared to only 1.13 dB in the control group (*p* < 0.0001). Complete hearing recovery was achieved in 13.15% of the ITS group vs. none in the control group. Multinomial logistic regression revealed a significantly higher probability of hearing recovery in the ITS group, with a relative risk of 8.52 [95% confidence interval (CI): 1.04–70.61; *p* < 0.05].

Further trials described the outcomes of methylprednisolone injections as salvage therapy for patients with SSNHL who failed to recover following oral steroid treatment. In a 63-patient cohort, 28.6% achieved remission, defined by improvements in functional hearing level, PTA, or speech discrimination score, while 71.4% did not respond (*p* = 0.039). Frequency-specific analysis revealed that hearing improvement was significantly greater at low frequencies compared to higher ones (*p* = 0.027). Interestingly, the delay between symptom onset and initiation of ITS, which averaged around 35–39 days, was not significantly associated with treatment outcomes (*p* = 0.680) (15). Similar results were described by Ferri et al. (16) after a 20-day period of treatment. Therapy led to a complete recovery in 13 patients (23.6%), with a mean hearing gain of 36.2%. Partial recovery was experienced by 10 patients (18.2%), and six patients (10.9%) exhibited slight improvement. However, the time from symptom onset to initiation of IST significantly affected the outcomes: patients who began IST earlier had better recovery rates, with a median delay of 12 days in the complete recovery group compared to 34 days in the non-recovery group (*p* = 0.007). The severity of hearing loss also affected the results; patients with initial hearing loss greater than 90 dB experienced a much lower improvement rate of 7.2%, while those with losses between 50 and 90 dB experienced a 21.2% improvement, and individuals with milder losses between 30 and 50 dB achieved a 47.6% recovery rate (*p* = 0.06). Recovery was more significant at low frequencies, with 67.2% of patients showing an improvement greater than 30 dB, compared to 16.3% at 8 kHz. A 14-patient prospective trial by Raymundo et al. (17) also observed a significative PTA improvement with a mean hearing gain of 27.33 dB with significant improvement across all tested frequencies (*p* < 0.001). Speech recognition rate also improved significantly (*F*_2,26_ = 13.208, *p* < 0.001).

Different criteria for defining recovery were used in other trials. Dallan et al. (18) defined a cutoff of 18% improvement using a relative gain analysis via receiver operating characteristic (ROC) curve. They observed a 55.6% improvement in 27-patient cohort (15 of them). The average PTA moving from 79.9 ± 21.4 to 60.6 ± 24.9 dB. Univariate analysis revealed a significant association between tinnitus and better recovery (*p* = 0.01). Multivariate stepwise regression analysis confirmed tinnitus (*p* = 0.01) and shorter time to therapy (*p* = 0.01) as independent predictors of hearing improvement, with neutrophilia also showing a weaker but statistically significant correlation (*p* = 0.02).

Methylprednisolone was successfully tested in association with conventional vasodilator and thrombolytic therapy. In a 97-patient trial, 83 received intratympanic methylprednisolone perfusion plus conventional vasodilator and thrombolytic therapy, while 14 received only traditional therapy without additional steroids. The authors observed the best improvement in patients who received treatment within 15 days of disease onset. In this subgroup, they observed an overall effective rate of 62.5% vs. 14.3%, *p* = 0.006, with a better improvement for low-frequency threshold (19).

Intratympanic injections are not the only way to administer methylprednisolone. In a study conducted in Nanjing Drum Tower Hospital, a microcatheter was used for SSNHL patients who failed to respond to 10 days or more of systemic therapy. A total of 26 subjects received 0.5 mL/day and 40 mg/mL daily perfusion of methylprednisolone for 10 days through a microcatheter. They were compared to a control group of 23 patients who underwent a second round of non-steroidal conventional treatment. The experimental group showed a significantly greater PTA improvement 20.2 ± 15.6 dB vs. 9.2 ± 13.7 dB, *p* = 0.011. PTA improvement of ≥15 dB was 50% in the IMP group vs. 21.7% in the control group (*χ*^2^ = 4.194, *p* = 0.041), and increased to 61.9% when excluding patients whose treatment initiation was delayed beyond 60 days from symptom onset (*p* = 0.007). There was a significantly better hearing recovery at low frequencies compared to high frequencies (*p* = 0.046) (20).

#### 3.1.2 Dexamethasone

Choi et al. (21) conducted a retrospective study assessing the effectiveness of ITS therapy with dexamethasone in patients with profound SSNHL who failed initial systemic steroid therapy. A total of 103 patients were enrolled and, according to Siegel’s criteria, the authors observed a significant improvement following ITS, with a recovery rate of 20.4% vs. 10.4%, respectively (*p* = 0.041). The average hearing gain after salvage therapy was 20.83 ± 16.71 dB. Multiple regression analysis identified pre-salvage PTA (odds ratio 1.169, *p* = 0.001), diabetes [odds ratio (OR): 0.069, *p* = 0.040], and symptom duration (OR: 9.242 for treatment initiated within 4–7 days, *p* = 0.042) as significant predictors of hearing recovery.

Salvage ITS therapy with dexamethasone was effective in several other trials. Erdur et al. (22) observed a significant improvement in a group of 21 patients treated with intratympanic dexamethasone compared to the control group of subjects with SSNHL refractory to initial systemic steroid therapy. They observed a hearing improvement greater than 20 dB in 47.6% of the intratympanic group compared to only 10% of the control group (*p* = 0.002). The mean PTA improvement was significantly higher in the intratympanic group (19.9 ± 16.5 dB) than in the control group (4.76 ± 9.6 dB, *p* < 0.01). Both groups exhibited greater gains at lower frequencies than at higher frequencies.

Significant improvement in PTA after salvage dexamethasone ITS was also observed in a prospective study by Dispenza et al. (23). The experimental 36-people population showed an improvement from an average PTA of 59.6 ± 16.7 to 46.8 ± 17.9 dB, corresponding to a mean hearing gain of 12.8 ± 15.2 dB (*p* < 0.01), whereas no hearing improvement was observed in the 10-patient control group who refused ITS. The authors also observed that a previous systemic steroid therapy gives a better post-salvage PTA (44.1 ± 15.3 dB) compared to those who had not received steroids (68.7 ± 24.9 dB, *p* < 0.01). Additionally, patients without a smoking history achieved a significantly greater mean hearing gain (15.9 ± 16.0 dB) compared to smokers (3.3 ± 6.6 dB, *p* < 0.05).

Another retrospective trial involving 54 patients (25 treated with ITS using dexamethasone and 29 controls who refused ITS) was conducted by Salvador et al. (24). The authors reported a significantly poorer PTA compared to controls (66.7 ± 26.9 dB vs. 51.5 ± 19.1 dB, *p* = 0.019) before ITS. Hearing improvement, defined as a ≥10 dB PTA gain, was achieved in 40% of the ITS group vs. 13.8% of the control group (*p* = 0.035). With a significant mean hearing gain of 8.7 ± 9.8 dB compared to only 0.7 ± 2.0 dB in the control group (*p* < 0.001). Frequency-specific analysis revealed that the ITS group achieved significant hearing gains at all measured frequencies (0.5, 1, 2, and 4 kHz) compared to the control group (*p* < 0.001 at each frequency), with the most pronounced improvements observed at lower frequencies (10.1 ± 13.2 dB at 0.5 kHz and 9.2 ± 8.8 dB at 1 kHz). Logistic regression confirmed that partial recovery following systemic steroids increased the odds of response to ITS by 11-fold (OR = 11.0; 95% CI: 1.6–75.5; *p* = 0.015).

Wu et al. (25) conducted a trial administering intratympanic dexamethasone as a salvage therapy for 180 patients with refractory SSNHL. They considered several outcomes discussed in the following paragraph, including the optimal timing and comparison with systemic salvage therapy. In this section, we report that there was a significant improvement in the ITS group with a PTA decrease of 29.2 ± 22.7 decibel hearing level (dB HL), and the best outcomes were observed when treatment began within 3 weeks (13.7 ± 15.8 dB HL vs. 6.1 ± 14.0 dB HL, *p* = 0.016) and even after 5 weeks (12.2 ± 18.7 dB HL vs. −1.6 ± 12.2 dB HL, *p* = 0.013). However, no significant difference was detected in salvage initiation between three and 5 weeks (*p* = 0.216).

The efficacy of dexamethasone was also confirmed in a randomized, double-blind, placebo-controlled trial. A total of 27 patients received intratympanic dexamethasone and 28 intratympanic saline injections. The dexamethasone group showed a significant mean improvement of 9.7 ± 8.5 dB in PTA compared to an improvement of only 4.5 ± 6.5 dB in the intratympanic normal saline injection (ITNI) group (*p* = 0.013). The ITS group achieved a PTA improvement of at least 15 dB by 29.6% vs. 7.1% in the placebo group (*p* = 0.032). Although 14.8% ITS patients experienced gains of ≥20 dB compared to only 3.6% in the placebo group, this difference did not reach statistical significance (*p* = 0.151). Dizziness was associated with poorer outcomes (*p* = 0.067) (26).

Lee et al. (27) stratified patients with refractory SSNHL by severity in two groups: 16 with severe hearing loss (70–90 dB) and 18 with profound SSNHL (greater than 90 dB) refractory to systemic therapy. After ITS, they observed recovery in six patients of the severe group and one in the profound group (37.5 and 5.5%, respectively).

ITS therapy using dexamethasone has also been evaluated in trials involving delayed treatment for refractory SSNHL. Li and Bennett (28) administered ITS with a median delay of 52 days (range: 14–81 days) from symptom onset to the first salvage treatment. They reported partial hearing recovery in only 1 out of 15 patients (6.7%), with no improvement in the remaining 14 patients (93.3%). The authors reviewed existing literature and concluded that the previously reported average recovery rate of 31.1% following ITS was likely influenced by earlier treatment initiation, as comparison studies had a median time to treatment of approximately 10–14 days, significantly shorter than in their study.

#### 3.1.3 Triamcinolone

Intratympanic triamcinolone acetonide was tested in one trial only involving 152 patients with refractory SSNHL after systemic corticosteroid therapy. The authors observed that the average hearing improvement following treatment was 15.9 ± 18.9 dB. Complete recovery, defined as a final PTA of within 10 dB of the unaffected ear, was achieved in 9.9% of patients, while 48% showed partial recovery, and 42.1% showed no improvement (29).

#### 3.1.4 Timing for ITS

The timing of intervention influenced outcomes across several studies. Raymundo et al. (17) observed that patients starting ITS between 14 and 21 days achieved a 90% recovery rate, while the success rate dropped to 33% if started between 21 and 28 days, and no recovery was observed if initiated after 28 days. Late ITS salvage therapy was also confirmed to have a poorer effect, as noted by Li and Bennet (28). The authors observed a 6.7% response rate in their population treated with a median delay between symptom onset and first salvage treatment of 52 days. And concluded that early delivery of salvage ITS could be the cause of their worse response compared to the literature (31.1%).

Wu et al. (25) conducted a trial with 270 profound SSNHL with poor response to initial systemic steroid therapy. They divided them into two groups: a group of 180 people receiving ITS with dexamethasone and 90 with hyperbaric oxygen therapy, *Ginkgo biloba* extract, and mecobalamin. In the ITS group, patients treated within 3 weeks showed the most significant improvement in low-frequency hearing (PTA decrease: 29.2 ± 22.7 dB HL) compared to the HY group (19.0 ± 18.7 dB HL; *p* = 0.021). For salvage initiated between 3–5 weeks, ITS still outperformed standard medical treatment (SMT) (22.2 ± 20.7 dB HL vs. 5.4 ± 19.2 dB HL; *p* < 0.001). Even after 5 weeks, ITS remained effective (20.1 ± 16.5 dB HL vs. 4.5 ± 12.4 dB HL; *p* < 0.001). In the high-frequency band, ITS showed significantly better outcomes when started within 3 weeks (13.7 ± 15.8 dB HL vs. 6.1 ± 14.0 dB HL; *p* = 0.016) and after 5 weeks (12.2 ± 18.7 dB HL vs. –1.6 ± 12.2 dB HL; *p* = 0.013), but not between 3–5 weeks (*p* = 0.216). Across all frequency bands, ITS consistently outperformed SMT (all *p*-values <0.001). A 50% clinical significance rate (>15 dB HL improvement) in the low-frequency band required SMT within 3 weeks. In contrast, ITS achieved similar rates even when started after 5 weeks, highlighting its broader therapeutic window. Patients treated with ITS experienced significantly greater improvement in hearing thresholds across all frequency bands compared to those receiving HY (all *p*-values <0.001).

#### 3.1.5 Primary vs. salvage ITS

Lan et al. (30) conducted a prospective randomized controlled trial to compare primary vs. salvage ITS in patients with refractory SSNHL. A total of 31 patients had primary ITS combined with systemic therapy, and 30 had salvage ITS after no response to standard treatment. No significant differences were observed after a 2-week follow-up with a PTA improvement of 29.9 ± 24.1 dB in the primary group and 28.3 ± 21.4 dB in the salvage group (*p* = 0.734), and the speech discrimination score improvements were 25.9 ± 32.5% and 24.0 ± 27.1%, respectively (*p* = 0.761). The early recovery rates, defined as a complete or a partial recovery, were also similar in the two groups over the first 2 weeks: 58.1% for the primary group and 60.0% for the salvage group (*p* = 1.000). At the three-month follow-up, the mean PTA improvement was 38.5 ± 22.0 dB in the primary group and 36.8 ± 22.3 dB in the salvage group (*p* = 0.762), and the SDS improvements were 34.3 ± 30.6% and 31.9 ± 27.9%, respectively (*p* = 0.659). A complete or a partial recovery was achieved in 67.7% of patients in the primary group and 73.3% in the salvage group (*p* = 0.780). The authors concluded that primary and salvage ITS provided equivalent hearing outcomes in SSNHL. Therefore, ITS salvage treatment should be preferred to avoid unnecessary injections, particularly in patients who already show early recovery after systemic therapy.

#### 3.1.6 Dexamethasone vs. methylprednisolone

Methylprednisolone and dexamethasone were compared as a salvage ITS treatment for SSNHL. Following Siegel’s criteria, complete recovery was achieved in 12% of patients, 48% showed partial or slight improvement, and 32% showed no recovery in the dexamethasone group. In contrast, in the methylprednisolone group, complete recovery was observed in 24% of patients, with an overall improvement rate of 84% compared to 64% in the dexamethasone group—a statistically significant difference (*p* < 0.05). The study concluded that intratympanic methylprednisolone is a more effective treatment compared to dexamethasone for SSNHL who do not respond to initial systemic therapy (31).

#### 3.1.7 Best ITS delivery strategy

Wang et al. (32) conducted a nonrandomized retrospective study to compare intratympanic dexamethasone delivery systems for refractory SSNHL. A total of 21 patients received continuous perfusion through a round window catheter, 23 transtympanic injections, 11 through a ventilation tube, and 32 patients who refused to receive medication were considered the control group. Average PTA improvement was 9.0 dB in the round window catheter group, 8.6 dB in the injections group, 1.7 dB in the ventilation tube group, and 1.4 dB in the control group. The first two groups showed significantly different improvements compared to the control group (*p* < 0.05), whereas the ventilation tube group had a similar outcome (*p* > 0.05). The authors considered a significant hearing improvement as a PTA gain >15 dB, they observed it in 38.1% of the continuous perfusion group (mean 29.8 dB), 34.8% in the injection group (29.4 dB), 9.1% in the ventilation group (26 dB) and 9.4% in control group (14.9%). The authors concluded that both round window catheter and repeated transtympanic injections are effective delivery systems for ITS, trans tympanic catheter, and no treatment have similar outcomes.

Ventilation tube tested when associated with a micropump for continuous steroid perfusion demonstrated different outcomes in refractory SSNHL. In a trial comparing results with those of a control group treated with standard transtympanic intermittent ITS, both groups received systemic corticosteroids concurrently. Michiba et al. (33) observed a mean hearing gain significantly higher in the experimental group (24.6 ± 14.1 dB vs. 16.6 ± 14.9 dB, *p* < 0.05). According to Siegel’s criteria, the response rate—defined as hearing improvement of 15 dB or more—was 70.0% in the experimental group and 46.7% in the ITS group, also reaching statistical significance (*p* < 0.05). The study concluded that continuous steroid perfusion via ventilation tube provides significantly better hearing recovery compared to intermittent intratympanic injections in patients with refractory SSNHL, particularly at low frequencies.

#### 3.1.8 Round window dexamethasone-releasing implants

Preliminary efficacy of biodegradable dexamethasone-releasing implants was tested. After a median follow-up of 217 ± 62 days post-implantation, the mean hearing threshold improved significantly by 31 ± 31 dB HL *p* < 0.05, paired *t*-test. Two out of the five patients (40%) achieved complete hearing recovery, defined as a return to normal hearing thresholds, while one patient (20%) showed partial recovery sufficient to restore serviceable hearing with 100% speech discrimination at amplified levels. The remaining two patients, both presenting with profound anacusis before treatment, showed no measurable improvement in hearing (10).

#### 3.1.9 Association of ITS and prophylactic migraine medications

ITS were tested in association with migraine prophylactic therapy in patients with SSNHL with an onset more than 6 weeks before evaluation. A total of 21 patients were enrolled and received nortriptyline, topiramate, and/or verapamil, along with lifestyle modifications; additionally, 71% of patients received intratympanic dexamethasone injections. Mean hearing threshold improved at 500 Hz from 55 ± 20 dB to 49 ± 19 dB (*p* = 0.01), and at 1,000 Hz from 57 ± 21 to 52 ± 19 dB (*p* = 0.03), low-frequency PTA improved from 57 ± 17 to 53 ± 15 dB (*p* = 0.01), and the speech-frequency from 60 ± 15 to 57 ± 13 dB (*p* = 0.02). The word recognition score showed a particularly strong response, improving from 45 ± 28% before treatment to 70 ± 28% after treatment (*p* < 0.01), and speech recognition threshold from 57 ± 18 to 50 ± 16 dB (*p* = 0.01). Clinically, ≥15% improvement in word recognition was observed in 68% of patients, and ≥10 dB improvement in speech recognition threshold was observed in 40%. Mild side effects from the medication were reported, including fatigue (24%), nausea/lightheadedness (10%), and dry mouth (10%) (7).

#### 3.1.10 Comparison between ITS and diuretics

Morita et al. (34) retrospectively compared the outcome of ITS, diuretics, and no intervention in a group of patients with idiopathic sudden sensorineural hearing loss (ISSNHL) after failure of initial therapy in a population affected by acute sensorineural hearing loss (2,000, 4,000, and 8,000 Hz ≤60 dB). Patients were initially treated with oral prednisolone, isosorbide, vitamin B12, and adenosine triphosphate disodium. After 14–16 days, non-responders were divided into three groups: those who underwent ITS (*n* = 27), the isosorbide one (*n* = 39), and a control group (*n* = 24). ITS had the highest recovery rates among groups (ITS vs. isosorbide vs. control: 77.8, 46.2, and 33.3% at 1 month; 70.4, 33.3, and 20.8, at 1 year; 33.3, 26.7, and 25.0% at 5 years, respectively). Those differences were statistically significant for ITS vs. diuretics at 1 month (*p* = 0.012) and 1 year (*p* = 0.006), and for ITS vs. controls at 1 month (*p* = 0.002) and 1 year (*p* < 0.001). However, no statistically significant differences were observed between groups at the 5-year follow-up. In terms of the PTA the mean gain was 53.3 dB at 1 month and 50.4 dB at 1 year for the ITS group, 24.1 and 18.9 dB for the diuretics group, and 17.7 and 15.0 dB for the control group.

### 3.2 Hyperbaric oxygen therapy

HY has been widely studied for refractory SSNHL treatment. Ajduk et al. (35) published in 2017 a retrospective study on patients who failed systemic steroid therapy, aiming to evaluate the effect of HY as salvage treatment. Forty-three subjects underwent HY and 50 served as a control group without additional treatment. Patients with hearing loss greater than 60 dB exhibited significant hearing improvement across all tested frequencies (*p*-values <0.01 at each frequency), while patients with milder hearing loss (≤60 dB) showed significant improvement only at the lower frequencies of 250 and 500 Hz. In contrast, patients in the control group exhibited no significant changes in hearing thresholds 1 month after steroid therapy failure. A similar trial design was conducted by Pezzoli et al. (36) in 23 patients and compared the outcomes with a 21-patient control group who declined HY. The mean PTA improvement was 15.6 ± 15.3 dB in the experimental group and 5.0 dB ± 11.4 in the control group, with the difference reaching statistical significance (*p* = 0.01). Recovery rates were significantly higher for patients treated with HY (69.6% vs. 19.1%, *p* = 0.001). Multivariable logistic regression confirmed that receiving HY was significantly associated with better recovery outcomes (OR: 33.6, 95% CI: 3.10–364.80, *p* = 0.004), particularly in achieving fair recovery (adjusted OR: 273.7, 95% CI: 6.10–12275.89, *p* = 0.004).

HY has been demonstrated to be effective for refractory SSNHL across multiple studies. Hosokawa et al. (37) observed better hearing recovery after salvage HY when compared to no therapy in SSNHL. A total of 167 patients were retrospectively compared to a 160-patient historical control group. A total of 9.6% of patients achieved complete recovery, 9.6% showed good recovery (≥30 dB hearing improvement), and 26.9% showed fair recovery (10–30 dB improvement), yielding an overall hearing improvement rate of 46.1%, compared to 32.5% in the control group (*p* = 0.021). The hearing recovery rate was relatively consistent across different grades of initial hearing loss. The timing of hyperbaric oxygen therapy (HBOT) initiation showed a potential effect, with patients starting within 7 days of steroid failure achieving a 63.2% recovery rate compared to 43.9% among those who began treatment later; however, this difference was not statistically significant (*p* = 0.144). Multiple logistic regression analysis confirmed that receiving HBOT was significantly associated with better hearing recovery, with an adjusted OR of 1.82 (95% CI: 1.09–3.02; *p* = 0.021). Conversely, the presence of vertigo was identified as a negative prognostic factor for hearing recovery (adjusted OR: 0.54; 95% CI: 0.31–0.96; *p* = 0.034). Muzzi et al. (38) observed an average hearing gain of 8.64 dB, corresponding to a relative average improvement of 16% in a population of 19 people. Improvements were frequency-specific, with the highest relative gains seen at low frequencies: 30% at 250 and 23% at 500 Hz, compared to only 12% at 4 kHz and 4% at 8 kHz. Multivariate analysis revealed that shorter therapeutic delay (*p* = 0.026) and older age (*p* = 0.037) were statistically significant positive prognostic factors for hearing recovery, especially at low frequencies. Therapeutic delay also affected outcomes: patients treated within 15 days showed an 11.67 dB (17%) improvement, those treated between 15 and 30 days a 10.83 dB (16%) improvement, while patients treated after 30 days showed only a 5 dB (10%) improvement. Alimoglu and Inci (39) published a retrospective trial describing the effects of HY in 36 patients with refractory SSNHL. The PTA improvement was 10.55 ± 13.56 dB (*p* < 0.05). Frequency-specific analysis revealed mean gains of 16.66 ± 18.43 dB at 0.25 kHz, 16.94 ± 19.93 dB at 0.5 kHz, 12.63 ± 16.71 dB at 1 kHz, 7.36 ± 15.28 dB at 2 kHz, 5.27 ± 11.58 dB at 4 kHz, and 2.91 ± 12.44 dB at 8 kHz, with significant improvements at all frequencies except 8 kHz. According to Siegel’s criteria, complete recovery occurred in three patients (8.33%), partial recovery in one patient (2.7%), slight recovery in five patients (13.88%), and no improvement in 25 patients (69.44%). Notably, higher recovery gains were more pronounced at lower frequencies, with approximately 17 dB improvement at 0.25 and 0.5 kHz.

#### 3.2.1 Optimal HY protocol

A recent article by Kim et al. (40) aimed to determine the optimal HY protocol for SSNHL when used in conjunction with systemic and ITS. They prospectively evaluated 105 patients randomly divided into three groups: Group 1 received HBOT at 2.5 atmospheres absolute (ATA) for 1 h daily for 10 days, Group 2 received 2.5 ATA for 2 h, and Group 3 received 1.5 ATA for 1 h. All patients also received oral methylprednisolone and ITS using dexamethasone. At 3-month follow-up, the mean PTA improvement was 53.8 ± 16.0 dB in Group 1, 52.5 ± 18.0 dB in Group 2, and 36.5 ± 24.8 dB in Group 3, with statistically significant differences (*p* = 0.002). The word discrimination score was also significantly better in Groups 1 and 2 (72.7 and 76.0%, respectively) compared to Group 3 (53.9%; *p* = 0.034). Complete recovery was achieved in 36.4% of Group 1 and 44.1% of Group 2 compared to 12.5% of Group 3 (*p* = 0.016). The combined complete and partial recovery rate was significantly higher in the 2.5 ATA groups (Group 1: 57.6%, Group 2: 58.8%) than in the 1.5 ATA group (31.3%; *p* = 0.043). The study concluded that HY at 2.5 ATA, regardless of the 1 or 2-h duration, offers superior hearing recovery outcomes compared to lower-pressure HBOT. It recommended 2.5 ATA for 1 h daily over 10 sessions as the optimal protocol when combined with corticosteroids.

#### 3.2.2 Use of HY after ITS as a salvage treatment

The following combined systemic and ITS was more effective than no further treatment in a cohort of 18 patients compared to 66 controls. Although final PTA and word discrimination score were not significantly different between the groups (*p* = 0.301 and *p* = 0.640, respectively), the mean hearing gain was significantly greater in the HY group (16.8 ± 4.5 dB) compared to the control group (4.5 ± 1.0 dB, *p* = 0.015) 3 months after treatment (41).

#### 3.2.3 Comparison between HY and ITS

Several studies compared the efficacy of HY and ITS. Yang et al. (42) retrospectively compared HY, ITS, and their combination in 105 patients with refractory SSNHL: 35 received ITS, 22 received HY, 19 received both, and 29 received no salvage therapy. PTA improved significantly in all treatment groups vs. control group (*p* = 0.02, 0.036, and 0.003). The combined group showed the most significant mean PTA gain (22.5 ± 18.7 dB), followed by ITS (18.87 ± 21.66 dB) and HY (17.39 ± 18.2 dB), though differences among treatment arms were not statistically significant. Word recognition scores improved significantly only in the combined group compared to the control (*p* = 0.035). Using a ≥15 dB gain threshold, recovery rates were highest in the combined group (68.4%), followed by ITS (48.6%), HY (45.5%), and control (22.2%) groups, with overall group differences significant (*p* = 0.018). ITS and combined groups had significantly higher recovery rates than the control (*p* = 0.033 and *p* = 0.002). Frequency analysis revealed greater hearing gains at lower frequencies, particularly at 250 Hz, where the combined group outperformed both ITS and HY (*p* = 0.044 and 0.021). The authors concluded that ITS, HY, and especially their combination, offer benefits over no salvage therapy, though larger prospective trials are needed. No statistically significant difference between ITS and HY was also reported by Ajduk et al. (35) in their retrospective study comparing 30 patients treated with ITS and 27 with HY for SNNHL nonresponsive to systemic steroid therapy. The mean hearing gain was 20.20 ± 19.77 dB in the ITS group and 12.81 ± 13.31 dB in the HY group (*p* = 0.217). Speech discrimination scores also improved in both groups, increasing by 16.13 ± 22.76 in the ITS group and by 8.59 ± 16.14 in the hyperbaric oxygenation (HBO) group (*p* = 0.113). The study concluded that ITS and HBO therapy produced similar hearing improvements in patients with refractory SSNHL. However, the small sample size limited the ability to detect potentially meaningful differences, and larger randomized controlled trials were recommended to confirm these findings.

Mariani et al. (43) confirmed a non-significant difference. In their retrospective study, they treated 34 patients with systemic steroids, 16 patients had systemic therapy in association with HY, and 12 with ITS. The authors observed no significant difference among groups in terms of PTA and hearing gain (60 ± 31.7 dB, 61.5 ± 20 dB, and 80.7 ± 29 dB, respectively, *p* = 0.1; 17.4 ± 15.4, 18.6 ± 21.1 and 15.7 ± 14.2 dB, respectively, *p* = 0.9). Therefore, the authors concluded that the association of HY or ITS to systemic therapy did not significantly improve hearing recovery in SSNHL patients who did not respond to systemic steroid therapy.

The larger study comparing ITS and HY was conducted by Wu et al. (25). A group of 180 people receiving ITS with dexamethasone was compared to 90 patients in the treatment arm receiving HY, *Ginkgo biloba* extract, and mecobalamin. Patients treated with ITS experienced significantly greater improvement in hearing thresholds across all frequency bands (25.1 ± 21.4 dB HL in the low-frequency band, 17.0 ± 16.6 dB HL in the language-frequency band, 15.8 ± 15.9 dB HL in the language plus 4 kHz band, and 11.4 ± 15.9 dB HL in the high-frequency band) (all *p*-values <0.001), vs. 10.5 ± 19.0, 7.7 ± 14.8, 7.2 ± 13.8, and 4.3 ± 12.8 dB HL. Clinically significant hearing recovery (defined as ≥15 dB HL improvement) was achieved in 62.2% of the ITS group vs. 42.2% in the SMT group for the low-frequency band (*p* = 0.003), 51.7% vs. 37.8% in the language-frequency band (*p* = 0.043), and 48.9% vs. 33.3% in the language plus 4 kHz band (*p* = 0.022). Differences in the high-frequency band (33.3% vs. 22.2%) were not statistically significant (*p* = 0.081). While both protocols showed better results when initiated earlier, the SMT group exhibited a sharp decline in effectiveness beyond 3 weeks post-onset, with minimal benefit observed after 5 weeks. Conversely, ITS maintained a notable degree of efficacy even when started more than 5 weeks after disease onset. Furthermore, they also observed that in the case of late intervention (after 5 or more weeks from diagnosis), patients treated with ITS experienced significantly greater improvement in hearing thresholds across all frequency bands compared to those receiving SMT (all *p*-values <0.001). A similar study design was also adopted by Ajduk et al. (44) in their retrospective study for SSNHL after failed systemic steroid therapy. A total of 43 patients received ITS, 35 received HY, and 48 received no additional treatment. Both the ITS and HY groups had a significant hearing recovery compared to controls (13.6 and 7.4 dB, respectively; *p* = 0.001). Additionally, 60.5% of patients treated with ITS and 42.9% treated with HY achieved significant hearing recovery. ITS also demonstrated a positive effect on tinnitus reduction, showing a statistically significant advantage over HY and observation (*p* = 0.002, OR 3.5). The presence of tinnitus before therapy was negatively correlated with hearing improvement, resulting in a 4.67 dB reduction in the average gain. Moreover, binary logistic regression analysis confirmed the superior efficacy of both ITS (*p* = 0.002, OR 30.28) and HY (*p* = 0.005, OR 22.18) compared to no treatment. The efficacy of HY and ITS was also compared in refractory high-frequency SSNHL by Sun et al. (45). A total of 31 patients received ITS, 32 patients received HY, and 41 patients received no salvage therapy, serving as a control group. After 1 month, the total effective rate for hearing recovery was 12.9% in the ITS group, 6.3% in the HY group, and 2.4% in the control group, but these differences were not statistically significant (*p* = 0.368 between ITS and HY; *p* = 0.197 between ITS and control; *p* = 0.809 between HBO and control). Regarding frequency-specific hearing gains, both the ITS and HY groups showed significant improvements at 2, 4, and 8 kHz when compared to baseline within each group. However, when comparing between groups, no significant differences were found between ITS and HY or between each salvage group and controls at 2 and 4 kHz (*p* = 0.468 and 0.934, respectively, for ITS vs. HY). At 8 kHz, ITS therapy produced significantly greater hearing gains than HY therapy (*p* = 0.049) and compared to the control (*p* = 0.025). In contrast, no significant difference was observed between the HBO and control groups (*p* = 0.873). Mean hearing gains at 8 kHz were 8.4 dB in the ITS group and 5.0 dB in the HY group. The authors concluded that while both ITS and HY therapies provided some degree of salvage benefit, ITS therapy demonstrated superior outcomes for tinnitus improvement and hearing recovery at 8 kHz compared to HBO therapy, and therefore should be considered the preferred salvage option for refractory high-frequency SSNHL.

### 3.3 Urokinase injection

New experimental therapies were tested for SSNHL unresponsive to conventional treatment such as intra-arterial pulsed injection urokinase (IAPU) through vertebral and external carotid arteries. Cui et al. (46) published a retrospective trial in which they treated 29 patients with IAPU and 38 with conventional protocols. PTA improved significantly better in IAPU group than in controls 34.2 ± 23.5 dB vs. 10.7 ± 13.1 dB; *p* < 0.001. Additionally, hearing recovery rates were higher in the IAPU group, with total, partial, and mild recovery observed in 20.7, 24.1, and 27.6% of patients, respectively, compared to 5.3, 10.5, and 13.2% in the control group. Notably, 27.6% of patients in the IAPU group showed no recovery, vs. 71.1% in the control group.

### 3.4 Explorative tympanotomy and round window-based therapies

Kampfner et al. (47) conducted a retrospective study to evaluate the efficacy of tympanotomy with sealing of the round window membrane in patients with severe to profound SSNHL who had failed to recover after conservative treatment. They observed a significant improvement in PTA after ear packing removal of 21.7 dB (*p* < 0.001). In a subgroup of 33 patients with later follow-up audiograms (mean 47.2 days after surgery), an additional improvement of 13.4 dB was recorded, resulting in a cumulative hearing gain of 32.8 dB compared to baseline (*p* = 0.0002). Patients aged 50 years or younger experienced significantly greater hearing recovery, with an average gain of 28.4 dB compared to 13.8 dB in older patients (*p* = 0.01). Age was inversely correlated with hearing improvement (Pearson’s *r* = −0.237, *p* = 0.021). According to Siegel’s criteria, complete recovery was achieved in 7% of patients, partial recovery in 13%, slight improvement in 36%, and no improvement in 28%, while deterioration occurred in 16%.

Loader et al. (48) used a triamcinolone-soaked fascia for sealing of the round window. A total of 25 patients were enrolled, and the authors observed a postoperative hearing gain of 20.4 dB (*p* = 0.0002). As observed by Kampfner et al. (47), the success of surgery was associated with age, as patients who improved had a mean age of 48.5 years compared to 61.8 years in those who did not (*p* = 0.004). Furthermore, no patient over the age of 65 demonstrated audiometric improvement following the procedure. The time to surgery was not statistically significantly associated with hearing outcomes (*p* = 0.21 in univariate analysis, *p* = 0.09 after adjustment for age). Linear regression analysis indicated that with every year increase in age, the postoperative hearing gain decreased by approximately 1 dB (*p* = 0.03).

Another strategy was proposed by Si et al. (9) in their randomized controlled study to assess round window niche drilling combined with intratympanic methylprednisolone in 20 SSNHL patients unresponsive to systemic and standard ITS. Patients were randomized into an experimental group or a control group receiving ITS alone. After 1 month, the experimental group showed significantly greater mean PTA improvement (20.38 ± 6.33 dB vs. 2.11 ± 1.07 dB; *p* = 0.004) and SDS improvement (19.3 ± 5.2% vs. 2.0 ± 0.82%; *p* = 0.004). Marked recovery (>30 dB gain) was seen in 40% of the experimental group vs. 0% of controls (*p* = 0.0867), while overall improvement (>15 dB gain) occurred in 50% vs. 0% (*p* = 0.0325). THI scores dropped by 33.9 points in the experimental group vs. 0.22 in controls (*p* < 0.001), and VAS scores decreased by 2.75 vs. 0.22 points (*p* = 0.001).

The round window membrane was also used as a delivery site for human IGF-1 delivered via gelatin hydrogels. Retrospective data from 25 patients were described by Nakagawa et al. (11). The mean baseline PTA threshold was 81.2 dB (95% CI: 71.2–91.1 dB), which improved significantly to 69.3 dB (95% CI: 59.8–78.7 dB) at 24 weeks after treatment (*p* < 0.001). Comparison with a historical cohort of patients treated with HY showed that the average hearing recovery in the IGF-1 group (11.9 ± 2.9 dB) was greater than that in the HBO group (8.0 ± 0.9 dB). However, this difference did not reach statistical significance (*p* = 0.08) except for 1 kHz pure tones (*p* = 0.04).

### 3.5 Constraint-induced sound therapy

Neurorehabilitative intervention could improve the outcomes of SSNHL. Sekiva et al. (49) administered constraint-induced sound therapy (CIST), a therapy consisting of plugging the intact ear and delivering music to the affected ear through a closed headphone for 6 h daily during hospitalization, aiming to reduce maladaptive cortical reorganization. A total of 22 patients received CIST and standard corticosteroid therapy alone, and 31 received systemic treatment only. At discharge, the hearing threshold difference between affected and intact ears was significantly smaller in the CIST + SCT group compared to SCT alone (*p* < 0.05, Bonferroni-corrected).

### 3.6 Statistical evaluation

**Figure 3:**
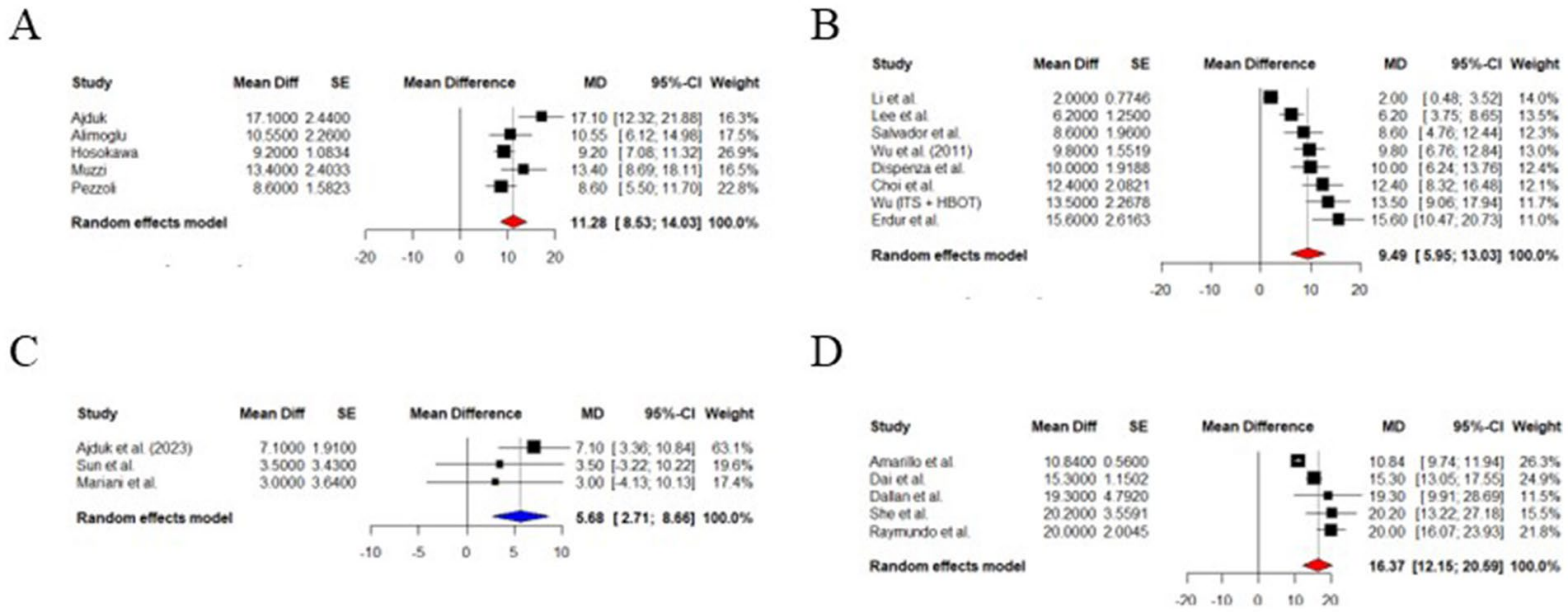
Forest plot. **(A)** PTA improvement after methylprednisolone treatment. **(B)** PTA improvement after dexamethasone treatment. **(C)** PTA improvement after HY treatment. **(D)** PTA improvement ITS vs. HY.

Meta-analysis was conducted among study groups, and data, and the results were summarized in Figure 3. The pooled analysis revealed a mean improvement in PTA of 16.37 dB (95% CI: 12.15–20.59 dB), which is statistically significant (*z* = 7.60, *p* < 0.0001) using a random-effects model for methylprednisolone ITS. There was a significant heterogeneity among studies *I*^2^ = 88.7% (95% CI: 76.3–94.6%), *τ*^2^ = 17.32, and a highly significant *Q*-test (*p* < 0.0001) (14, 17–20). The forest plot is depicted in Figure 3.

Random effects model estimated a mean PTA improvement of 9.94 dB (95% CI: 5.95–13.03 dB), which is statistically significant (*z* = 5.25, *p* < 0.0001) for dexamethasone as ITS for SSNHL nonresponding to systemic corticosteroids. There was considerable variability between studies *I*^2^ = 90.3% with the *Q*-test for heterogeneity being highly significant (*p* < 0.0001), and *τ*^2^ = 22.67 (21–28). Forest plot summarizing outcomes is included in Figure 3 (21–28).

HY was also effective in a meta-analytic study using a random-effects model. It showed a PTA improvement of 11.28 dB (95% CI: 8.53 to 14.03 dB), a result was statistically significant (*z* = 8.04; *p* < 0.0001). The heterogeneity between studies was moderate to substantial (*I*^2^ = 65.6%; *τ*^2^ = 6.15, and a *Q*-test *p*-value = 0.0204). This result confirms the beneficial salvage therapy for refractory SSNH using HY (Figure 3) (35–39).

ITS led to a better mean PTA improvement than HY alone (5.68 dB greater; 95% CI: 2.71 to 8.66 dB), with a statistically significant difference (*z* = 3.75, *p* = 0.0002). This finding has high consistency due to the absence of detectable heterogeneity (*I*^2^ = 0%) (35, 42–45).

### 3.7 Risk of bias

Risk of bias was assessed using the ROBINS-I tool. Most of the included studies had a retrospective and non-randomized design, which led to a risk of bias due to significant confounding, lack of randomization, and potential for selective reporting, as seen in studies by Li and Bennett (28), Yang et al. (42), and Muzzi et al. (38). In contrast, a minority of papers were randomized controlled trials, such as the one from Wu et al. (25), a double-blinded randomized controlled trial, provided high-quality evidence supporting the efficacy of intratympanic dexamethasone therapy. In contrast, Raymundo et al. (17), although prospective, lacked a control group and did not fully address potential confounders, resulting in a moderate risk of bias due to confounding and selection bias. Unclear reporting of intervention timing, incomplete outcome data, and possible selective outcome reporting led several papers to be judged as potentially affected by moderate risk of bias. For example, in the Dallan et al. (18) article, although a significant improvement in PTA was reported, the retrospective nature and lack of a control group raise concerns about the reliability of the observed effect. Similarly, Belhassen and Saliba (15) and Choi et al. (21) were both rated as serious risk in multiple domains, particularly due to potential confounding and limited information on adherence to protocol. In contrast, Amarillo et al. (14), though non-randomized, utilized a large sample size and included a control group, which improved its assessment to moderate risk, particularly due to better reporting on intervention classification and outcomes. This makes it a reliable observational study. These assessments indicate that while some studies provide high-quality, low-bias evidence, a significant proportion exhibit limitations that weaken the overall strength of the conclusions. Notably, bias due to confounding and lack of control groups was the most frequent issue. This variability in methodological rigor across studies contributes to the moderate-to-low certainty of evidence in pooled analyses. Accordingly, while our meta-analyses suggest a benefit from ITS and HBOT in refractory SSNHL, the strength of the evidence is tempered by the quality of contributing studies. Future research should prioritize robust randomized designs and consistent reporting of outcomes to enhance the reliability of comparative effectiveness data. A summary of the risk of bias assessment is presented in Figure 1 using the ROBVIS tool to enhance readability and facilitate visual interpretation for the reader.

## 4 Discussion

SSNHL affects up to 27 per 100,000 people annually (1, 2). After treatment, more than half of them attain incomplete recovery (50). Therefore, it is necessary to have a treatment protocol for patients suffering from SSNHL non-responsive to first-line therapy.

Both intratympanic methylprednisolone and dexamethasone have shown efficacy in treating SSNHL unresponsive to standard systemic therapy across multiple studies. Although most were not prospective randomized controlled trials, the consistency of positive outcomes supports their effectiveness. Methylprednisolone was commonly administered at a concentration of 40 mg/mL, although the timing and number of injections varied between studies (14–28). Dexamethasone was administered at varying concentrations, ranging from 0.5–5 mg/mL. Eight studies evaluated its use as salvage therapy for SSNHL, with positive outcomes reported in all but one. The exception was the study by Li and Bennett (28), which involved patients who began treatment after a prolonged delay, likely contributing to the limited therapeutic effect (21–26, 28). These findings were further supported by a double-blind, placebo-controlled trial, which provided more substantial evidence for the efficacy of dexamethasone, showing a significantly greater PTA improvement compared to the control group (*p* = 0.032) (26). Although both methylprednisolone and dexamethasone demonstrated improvements in PTA among patients with refractory SSNHL, the Grading of Recommendations Assessment, Development, and Evaluation (GRADE)-based assessment indicates a higher certainty of evidence for dexamethasone. This is primarily due to the randomized controlled trial by Wu et al. (26). In contrast, the evidence supporting methylprednisolone derives exclusively from non-randomized studies with a significant variation in effect size (PTA from ~10 to 27 dB). Similarly, the evidence for HY is also rated as low, as it shares many of the limitations with the methylprednisolone trials. These include reliance on observational, retrospective, or uncontrolled designs, variability in PTA improvements (6–17 dB), and non-uniformity among treatment protocols. Due to the heterogeneity among the included studies, we have summarized their key characteristics and outcomes in Table 1 to facilitate comparison.

**Table 1:** Intratympanic steroids trials for refractory SSNHL.

Study (author, year)	Study design	Criteria for refractory SSNHL	Population (*n*, age)	ITS treatment (dose/timing)	Results/Statistics
Dallan et al., 2010 (18)	Retrospective case series	No improvement after 7–10 days systemic steroids	*n* = 27; mean age 56.5 ± 15.6 years	1 mL MP 40 mg/mL + NaHCO₃, single injection	Mean PTA improvement: from 79.9 to 60.6 dB (*p* < 0.05); 55% showed “useful” improvement
Ferri et al., 2012 (16)	Prospective non-randomized	<50% recovery after 10 days IV betamethasone	*n* = 55; mean age 49.7 years	MP 40 mg/mL, 0.5 mL, up to 7 times in 20 days	52.7% improved; complete: 13 (36.2%), partial: 10 (18.9%), slight: 6 (16.2%)
Raymundo et al., 2010 (17)	Prospective study	No improvement after 10 days oral prednisolone	*n* = 14; mean age 43.8 years	MP 40 mg/mL, 0.3–0.5 mL, 3 injections on alternate days	71.4% improved (≥20 dB or ≥20% SRT); mean gain 27.3 dB among responders
She et al., 2010 (20)	Prospective controlled	<15 dB improvement after ≥10 days of standard treatment	*n* = 26 (SG), 23 (CG)	MP 40 mg/mL, 0.5 mL daily for 10 days via microcatheter	SG: 50% effective; PTA gain 20.2 ± 15.6 dB vs. 9.2 ± 13.7 dB in CG (*p* = 0.011); more effective if started ≤60 days
Belhassen and Saliba, 2014 (15)	Retrospective chart review	No recovery after oral steroids (≤2 months since onset)	*n* = 63	MP, up to 3 injections, 1/week	28.6% remission; PTA plateaued after 2nd injection; SRT improved after 3rd
Amarillo et al., 2022 (14)	Controlled study	Failure after systemic steroids	*n* = 76 treated, 125 total	MP 40 mg/mL, multiple injections (unspecified)	PTA gain: 10.84 vs. 1.13 dB (*p* < 0.0001); RR for recovery with ITS: 8.52 (CI 1.03–70.61)
Dai et al., 2017 (19)	Prospective controlled	Failure after standard treatment	*n* = 83 (39 with short interval)	MP, specifics not detailed; grouped by onset-to-treatment	61.5% effective vs. 20.5% (*p* < 0.001); greater low-frequency hearing gain; interval ≤15 days better outcomes
Choi et al., 2020 (21)	Retrospective case control	No/partial response after systemic steroids (per Siegel’s criteria)	*n* = 115; profound ISSNHL	Dexamethasone 5 mg/mL, 0.3–0.4 mL × 3 over 2 weeks	Serviceable hearing recovery: 20.4% vs. 10.4% (*p* = 0.041); key predictors: PTA, diabetes, symptom duration
Dispenza et al., 2013 (23)	Prospective	No improvement after systemic therapy (<10 dB PTA gain)	*n* = 36 treated, 10 control	Dexamethasone 4 mg/mL; 24.3 days mean delay from onset	Mean PTA improved from 59.6 to 46.8 dB; mean gain 12.8 dB; smokers responded worse (*p* < 0.05)
Erdur et al., 2014 (22)	Retrospective control	<20 dB improvement in PTA after systemic steroids (14 days)	*n* = 21 ITS, 30 control	Dexamethasone 1 mg/mL, drops 4×/day via tube for 2 weeks	PTA gain: 19.9 vs. 4.76 dB (*p* = 0.002); response: 47.6% vs. 10%
Lee et al., 2010 (27)	Retrospective	No recovery after systemic dexamethasone	*n* = 47; 25 severe, 22 profound	Dexamethasone 5 mg/mL, 0.3–0.4 mL, 6 injections over 2 weeks	Improvement in severe: 37.5%, profound: 5.5% (*p* = 0.03)
Li and Bennett, 2022 (28)	Retrospective	Salvage after failed prior treatment	*n* = 15	Dexamethasone 3.3 mg/mL; up to 3 weekly injections	Only 1/15 improved (6.7%); mean delay: 52 days
Salvador et al., 2021 (24)	Retrospective chart review	No complete recovery after systemic steroids	*n* = 54 (25 treated, 29 control)	Dexamethasone 4 mg/mL, 0.5–1 mL weekly, up to 4 injections	Improvement: 40% vs. 13.8% (*p* = 0.035); mean gain 8.6 vs. 0.7 dB
Wu et al., 2022 (25)	Retrospective, comparative	No improvement ≥2 weeks post systemic therapy	*n* = 270 (180 ITS, 90 SMT only)	Dexamethasone 5 mg/mL, 0.5 mL, 3×/week for ≥2 weeks	Greater hearing gain with ITS in all frequencies; best if started <3 weeks
Wu et al., 2011 (26)	Randomized, double-blind, placebo-controlled	No response to systemic steroids	*n* = 60 (55 completed)	ITS: Dexamethasone 0.5 mL, 4 injections in 2 weeks	PTA gain: 9.8 vs. 4.5 dB (*p* < 0.05); responders: 44.4% vs. 10.7%

MP, methylprednisolone; PTA, pure tone average; SRT, speech recognition threshold; SDS, speech discrimination score; SG/CG, study/comparison group.

The choice between dexamethasone and methylprednisolone as salvage treatments for SSNHL unresponsive to systemic therapy was directly compared. Both groups showed clinical improvement based on Siegel’s criteria; however, the methylprednisolone group demonstrated significantly greater improvement (*p* < 0.05) (31).

Another important consideration is the role of ITS as a primary treatment for SSNHL. Lan et al. (30) compared the efficacy of primary vs. salvage ITS and found no statistically significant difference between the two groups. Based on these findings, they recommended reserving ITS as a salvage therapy to avoid unnecessary injections, particularly in patients demonstrating early recovery following systemic steroid treatment.

HY was consistently associated with significant hearing improvement across all reviewed studies, particularly at lower frequencies (35, 37, 39). The only study reporting limited or no functional benefit was conducted by Alimoglu and Inci (39), which lacked a control group and assessed outcomes retrospectively using Siegel’s criteria (38). The recommended HY protocol involves 2.5 ATA pressure for 1–2 h per session, as lower pressures have been linked to poorer outcomes (40). However, when HY was administered after both systemic and intratympanic steroid treatments, no statistically significant improvement was observed in either pure-tone average (PTA) or word discrimination scores.

ITS, HY, and their combination all resulted in statistically significant improvements in PTA compared to the control group. Although the combined therapy achieved the greatest improvement, followed by ITS and then HY, the differences between these treatment groups were not statistically significant. Notably, word recognition scores improved significantly only in the combined therapy group compared to the control group (25, 35, 42). These findings have been corroborated by several other studies, except for Mariani et al. (43), who reported no significant difference when these therapies were administered alongside systemic steroids (35, 43). Across studies, ITS generally yielded better outcomes than HY, though most differences were not statistically significant. An exception was one trial where ITS showed significantly greater hearing gain than HY (43). The absence of a significant difference between ITS and HY has also been supported by systematic reviews and meta-analyses conducted by Kuo et al. (51) and Lin et al. (52). However, the combination of ITS and HY as a salvage treatment for refractory SSNHL is the best option according to Lin et al. (52).

Refractory SSNHL has a significant impact on quality of life. It is also worsened by the presence of tinnitus, which constitutes a frequent comorbidity that may persist and, with time, may become the patient’s primary concern (1, 33). ITS showed a significant advantage over HY and observation on tinnitus reduction (*p* = 0.002) (44) and in case of high frequency, SSNHL should be considered the preferred salvage option for refractory high-frequency SSNHL (45).

The timing of ITS salvage therapy appears to influence outcomes. Several studies observed that a delay in ITS therapy results in a poorer outcome compared to early treatment (14, 16, 19, 29). However, not all the authors observed that, for example, Belhassen and Saliba (15) did not notice any differences related to the timing of ITS administration.

Maladaptive cortical reorganization could be one of the causes of poor outcomes after SSNHL. CIST could reduce it and improve the outcomes of SSNHL (49). Given the noninvasive strategy of a therapy based on music listening, its use could be recommended during rehabilitation.

The remaining strategies, such as IGF1-injections using round window membrane, tympanotomy with the sealing of the round window membrane, urokinase injections, diuretics, prophylactic migraine medication, round window dexamethasone releasing implants, triamcinolone ITS, showed promising outcomes but need further studies to be confirmed in the current clinical practice.

**Figure 4:**
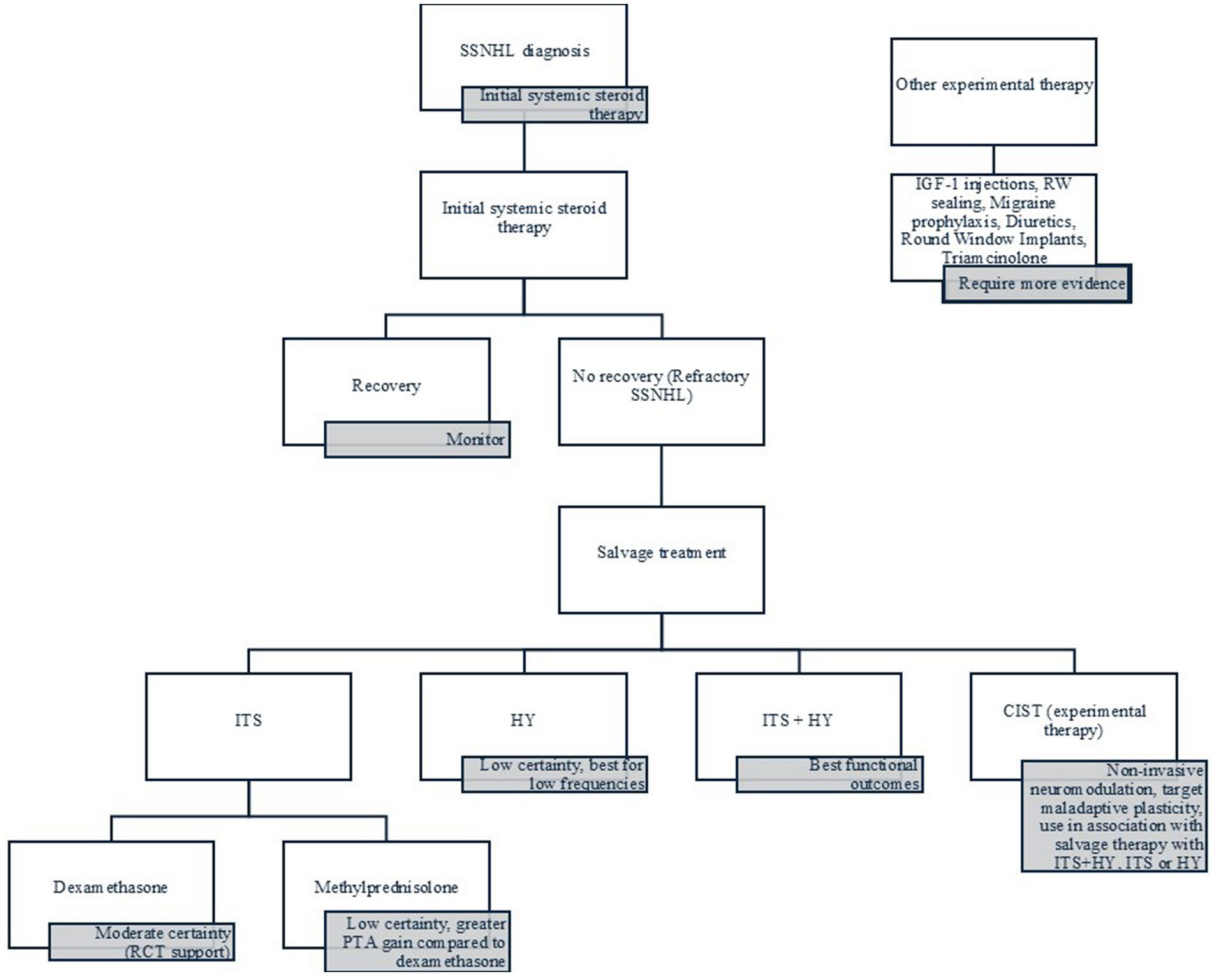
Treatment algorithm for refractory SSNHL.

Figure 4 summarizes the evidence in a practical flowchart for the clinical management of refractory SSNHL.

## 5 Conclusion

SSNHL remains a challenging condition, while spontaneous recovery is possible, more than half of patients experience incomplete improvement, highlighting the need for effective salvage treatment strategies. ITS with both methylprednisolone and dexamethasone demonstrated consistent efficacy across multiple studies, with methylprednisolone showing slightly better outcomes in some comparisons. HY also offers benefits, particularly at lower frequencies, and its combination with ITS appears to provide the most significant functional gain, especially in word recognition. Significantly, early initiation of ITS is generally associated with better outcomes, though some variability exists in the literature. ITS alone demonstrated better PTA improvement compared to HY-only therapy. For high-frequency SSNHL or persistent tinnitus, ITS may be the preferred option. Additionally, non-invasive neuromodulatory strategies such as coordinated reset therapy (CIST) show promise in addressing maladaptive cortical changes that may underlie poor recovery. While other emerging therapies—such as IGF-1 application, sealing of the round window, and migraine prophylaxis—show encouraging preliminary results, they require further validation through robust, randomized trials. Altogether, a stepwise, evidence-based approach tailored to the timing, severity, and symptom profile of SSNHL is essential to optimizing outcomes and minimizing long-term disability.

## Data Availability

The original contributions presented in the study are included in the article/Supplementary material, further inquiries can be directed to the corresponding authors.

## Footnotes

^1^ https://www.riskofbias.info/welcome
