# Supplementary material for: Salvage treatment strategies for refractory sudden sensorineural hearing loss—a comprehensive review and meta-analysis with practical recommendations
Source: Front Neurol. 2025 Jul 15;16:1627892. doi: 10.3389/fneur.2025.1627892 (PMC12305195; doi:10.3389/fneur.2025.1627892)
Supplement: Supplementary file 1 [file Supplementary_file_1.docx]

# Supplementary Material

## Supplementary Table 1. Search Strategy

| Database | Search Query | Results |
| --- | --- | --- |
| PubMed | ("single side deafness" OR "sudden sensorineural hearing loss") AND (rehabilitation OR recovery OR recuperation) [2010-2025, English only] | 799 |
| Embase | ('single side deafness' OR (single AND side AND ('deafness'/exp OR deafness))) AND ('rehabilitation'/exp OR rehabilitation OR 'recovery'/exp OR recovery OR recuperation) | 121 |
| Cochrane | single side deafness OR sudden sensorineural hearing loss OR hearing loss | 97 |
| Combined Search | All queries above aggregated | 1017 |
|  | Deduplication | 988 |

Screening Process

- Articles after title/abstract screening: 413

- Full-text screened and included in final review: 41

## PICOS

| **Component** |  |
| --- | --- |
| **P – Population** | Adults (> 18 years old) with sudden sensorineural hearing loss (SSNHL) refractory to initial systemic treatment |
| **I – Intervention** | Any therapeutic intervention for refractory SSNHL |
| **C – Comparator** | Placebo, Hyperbaric oxygen (HBO), no treatment, systemic steroids alone, or other salvage therapies |
| **O – Outcomes** | Improvement in pure-tone average (PTA), speech discrimination score (SDS), word recognition score (WRS), or rate of hearing recovery |
| **S – Study design** | Randomized controlled trials (RCTs), non-randomized controlled studies, cohort studies, and retrospective observational studies |

## Supplementary Table 2. Risk of Bias Assessment (ROBINS-I)

| Study (Author, Year) | Confounding | Selection of participants | Classification of interventions | Deviations from intended interventions | Missing data | Measurement of outcomes | Selection of reported result | Overall risk of bias |
| --- | --- | --- | --- | --- | --- | --- | --- | --- |
| Ajduk et al. (2017) | Moderate | Low | Low | Low | Low | Low | Moderate | Moderate |
| Ajduk et al. (2023) | Moderate | Low | Low | Low | Low | Low | Moderate | Moderate |
| Alimoglu and Inci (2016) | Serious | Moderate | Low | Low | Low | Low | Moderate | Serious |
| Amarillo et al. (2019) | Moderate | Low | Low | Low | Low | Low | Moderate | Moderate |
| Andrianakis et al. (2021) | Moderate | Low | Low | Low | Low | Low | Moderate | Moderate |
| Belhassen and Saliba (2014) | Moderate | Low | Low | Low | Low | Low | Moderate | Moderate |
| Berjis et al. (2016) | Low | Low | Low | Low | Low | Low | Low | Low |
| Choi et al. (2020) | Moderate | Low | Low | Low | Low | Low | Moderate | Moderate |
| Cui et al. (2021) | Moderate | Low | Low | Low | Low | Low | Moderate | Moderate |
| Dai et al. (2017) | Moderate | Low | Low | Low | Low | Low | Moderate | Moderate |
| Dallan et al. (2010) | Moderate | Low | Low | Low | Low | Low | Moderate | Moderate |
| Dispenza et al. (2013) | Moderate | Low | Low | Low | Low | Low | Moderate | Moderate |
| Erdur et al. (2013) | Moderate | Low | Low | Low | Low | Low | Moderate | Moderate |
| Ferri et al. (2012) | Moderate | Low | Low | Low | Low | Low | Moderate | Moderate |
| Goshtasbi et al. (2021) | Moderate | Low | Low | Low | Low | Low | Moderate | Moderate |
| Gülüstan et al. | Moderate | Low | Low | Low | Low | Low | Moderate | Moderate |
| Hosokawa et al. (2017) | Moderate | Low | Low | Low | Low | Low | Moderate | Moderate |
| Kampfner et al. (2013) | Moderate | Low | Low | Low | Moderate | Low | Moderate | Moderate |
| Kim et al. (2023) | Low | Low | Low | Low | Low | Low | Low | Low |
| Lan et al. (2023) | Low | Low | Low | Low | Low | Low | Low | Low |
| Lee et al. (2010) | Moderate | Low | Low | Low | Low | Low | Moderate | Moderate |
| Lee et al. (2024) | Moderate | Low | Low | Low | Low | Low | Moderate | Moderate |
| Li and Bennett (2022) | Serious | Moderate | Low | Low | Low | Low | Moderate | Serious |
| Loader et al. (2013) | Moderate | Low | Low | Low | Low | Low | Moderate | Moderate |
| Mariani et al. (2023) | Moderate | Low | Low | Low | Low | Low | Moderate | Moderate |
| Mingbao et al. (2024) | Moderate | Low | Low | Low | Low | Low | Moderate | Moderate |
| Morita et al., 2016 | Serious | Moderate | Low | Moderate | Low | Low | Moderate | Serious |
| Muzzi et al. | Serious | Moderate | Low | Low | Low | Low | Moderate | Serious |
| Nakagawa et al. | Serious | Low | Low | Low | Low | Low | Moderate | Serious |
| Pezzoli et al. (2015) | Moderate | Low | Low | Low | Low | Low | Moderate | Moderate |
| Plontke et al. | Serious | Moderate | Low | Low | Low | Low | Moderate | Serious |
| Raymundo et al. (2010) | Moderate | Low | Low | Low | Low | Low | Moderate | Moderate |
| Salvador et al. (2021) | Moderate | Low | Low | Low | Low | Low | Moderate | Moderate |
| Sekiya et al. (2016) | Serious | Moderate | Low | Low | Low | Low | Moderate | Serious |
| She et al. (2019) | Serious | Moderate | Low | Low | Low | Low | Moderate | Serious |
| Si et al. (2018) | Low | Low | Low | Low | Low | Low | Low | Low |
| Sun et al. (2018) | Moderate | Low | Low | Low | Low | Low | Low | Moderate |
| Wang et al. (2012) | Serious | Moderate | Low | Low | Low | Low | Moderate | Serious |
| Wu et al. (2011) | Low | Low | Low | Low | Low | Low | Low | Low |
| Wu et al. (2022) | Moderate | Low | Low | Low | Low | Low | Moderate | Moderate |
| Yang et al. (2013) | Serious | Moderate | Low | Low | Low | Low | Moderate | Serious |
